# Positive association between high-density lipoprotein cholesterol and bone mineral density in U.S. adults: the NHANES 2011–2018

**DOI:** 10.1186/s13018-022-02986-w

**Published:** 2022-02-15

**Authors:** Ruijie Xie, Xiongjie Huang, Qianlong Liu, Mingjiang Liu

**Affiliations:** 1 Department of Hand Surgery, The Affiliated Nanhua Hospital, Hengyang Medical School, University of South China, Hengyang, 421002 China; 2 Department of Hand Surgery, The Affiliated Changsha Central Hospital, Hengyang Medical School, University of South China, Changsha, 410004 China

**Keywords:** High-density lipoprotein cholesterol, Bone mineral density, Osteoporosis, Osteopenia

## Abstract

**Background:**

Serum lipids are highly inheritable and play a major role in bone health. However, the relationship between high-density lipoprotein cholesterol (HDL-C) and bone mineral density (BMD) remains uncertain. The goal of this study was to see if there was a link between HDL-C levels and BMD in persons aged 20–59.

**Methods:**

Multivariate logistic regression models were used to determine the link between HDL-C and lumbar BMD using data from the National Health and Nutrition Examination Survey (NHANES) 2011–2018. Generalized additive models and fitted smoothing curves were also used.

**Results:**

The analysis included a total of 10,635 adults. After controlling for various variables, we discovered that HDL-C was positively linked with lumbar BMD. The favorable connection of HDL-C with lumbar BMD was maintained in subgroup analyses stratified by sex and race in women, but not in men, and in blacks, but not in whites. The relationship between HDL-C and lumbar BMD in men and whites was a U-shaped curve with the same inflection point: 0.98 mmol/L.

**Conclusions:**

In people aged 20 to 59, our research discovered a positive relationship among HDL-C and lumbar BMD. Among males and whites, this relationship followed a U-shaped curve (inflection point: 0.98 mmol/L). HDL-C measurement might be used as a responsive biomarker for detecting osteoporosis early and guiding therapy.

## Background

Serum lipids are highly inheritable and play a major role in a variety of metabolic disorders [1]. Because high-density lipoproteins (HDL) help to transport excess cholesterol from peripheral locations to the liver and contain the cholesterol carried by these lipoproteins (HDL-C) is referred to as "beneficial cholesterol." HDL-C is regarded to be beneficial to health, with higher levels being better for long-term cardiovascular disease prevention [2].

Osteoporosis and cardiovascular diseases (CVDs) are two major public health issues around the world [3, 4]. Osteoporosis affects up to 49 million people in North America, Europe, Japan, and Australia. CVDs caused approximately 17.8 million deaths worldwide in 2017 [5, 6]. Both osteoporosis and cardiovascular diseases increase the risk of morbidity and mortality [7, 8].

Osteoporosis is a long-term disorder marked by reduced bone mineral density (BMD) that affects a huge number of people [9]. According to the International Osteoporosis Foundation, more than 30% of women and more than 20% of men over the age of 50 have osteoporosis or osteopenia, putting them at risk for osteoporotic fractures [10]. Simultaneously, the prevalence of osteoporosis continues to climb as the population ages and expands [11]. Apart from genetics, age, and gender, other variables that affect bone metabolisms, such as lipid metabolism and lifestyle, have lately received a lot of attention [12–14]. Meanwhile, scientists are working to discover novel ways to prevent and treat osteoporosis.

Cholesterol is now known to have an important role in bone metabolism, according to several research [15–17]. Recent research has focused on the link between HDL-C and BMD, although there have been conflicting results reported in this small body of evidence [18–23]. Most of these studies concluded that HDL-C levels were negatively associated with BMD, and this relationship was more pronounced in the population of menopausal women [18–21]. However, there are also some studies that suggest a positive correlation [22], or no correlation between HDL-C levels and BMD [23]. The findings of these researches are still up for debate. Since a result, it's worth investigating the relationship between HDL-C levels and BMD to see if HDL-C levels may be used to predict the likelihood of osteoporosis or osteopenia, as this might give a novel theoretical framework for understanding the genesis of the disease and designing therapies. As a result, we assessed the connection between HDL-C and BMD in this study using a comprehensive fraction of individuals aged 20 to 59 from the National Health and Nutrition Examination Survey (NHANES).

## Materials and methods

### Data source and study population

**Fig. 1:**
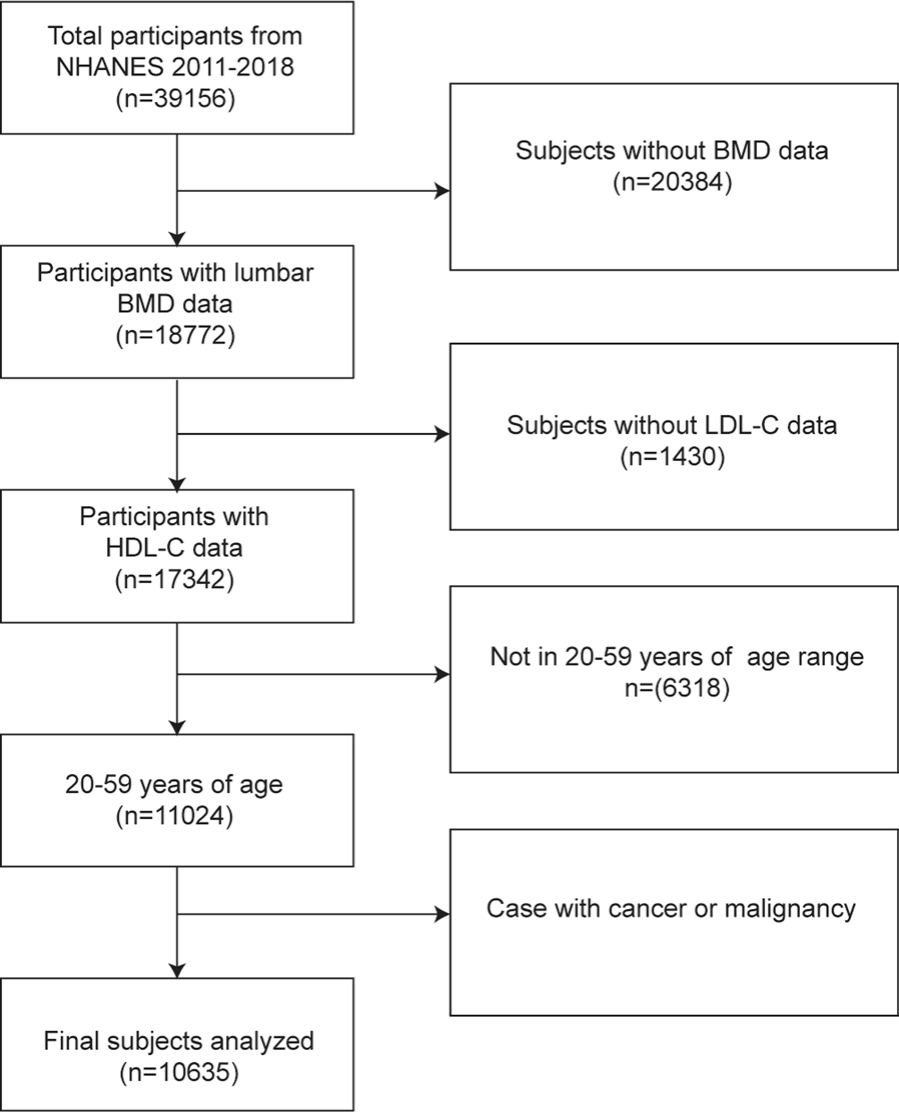
Flowchart of participants selection. NHANES, National Health and Nutrition Examination Survey; HDL-C, high-density lipoprotein cholesterol; *BMD* bone mineral density

The NHANES is a major, continuing cross-sectional survey in the United States that aims to give objective statistics on health issues and address emerging public health concerns among the general public. The National Center for Health Statistics' Institutional Review Board authorized the survey techniques, and all NHANES participants gave their agreement for their data to be utilized for the study. The NHANES datasets were utilized for this investigation from 2011 to 2018. The participants in the research had to be between the ages of 20 and 59. Among the 39,156 eligible adults, we excluded 20,384 individuals with missing BMD data, 1430 with missing HDL-C, 6318 individuals younger than 20 years or older than 59 years, and 389 individuals with cancer diagnoses. In the end, 10,635 people were enrolled in the study (Fig. 1).

### Study variables

**Fig. 2:**
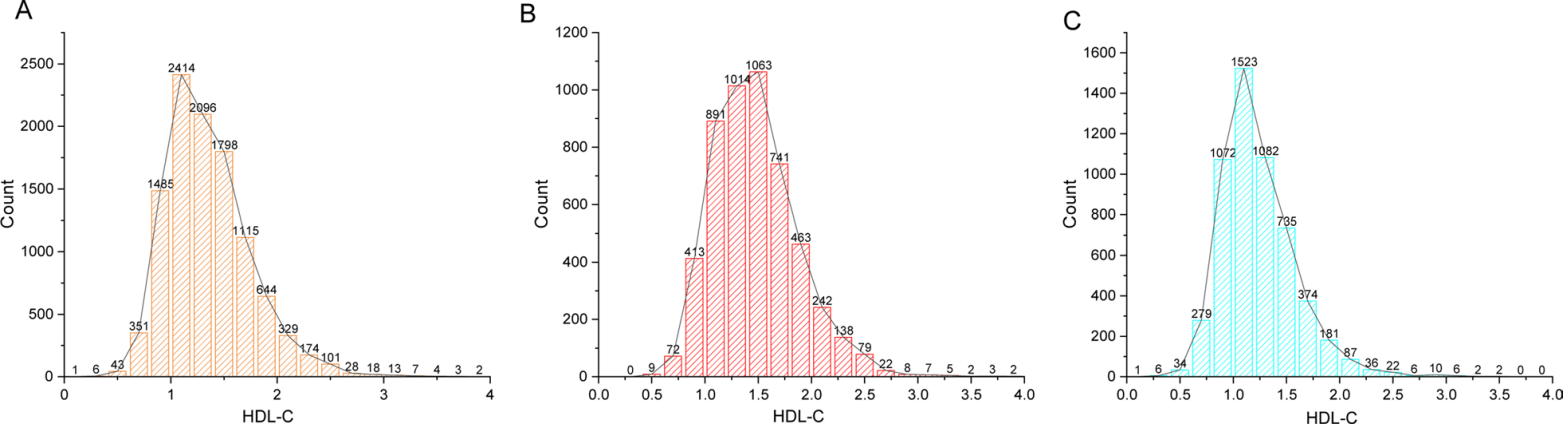
Distribution histogram of HDL-C. **A** Among all participants; **B** Among all males; **C** Among all females. HDL-C, high-density lipoprotein cholesterol

HDL-C was measured in a sample of serum. Under the test conditions, apoprotein-B (apoB)-containing lipoproteins were removed by blocking reagents and rendering them nonreactive with enzymatic cholesterol reagents. In the presence of magnesium ion, HDL-C levels were determined using polyethylene glycol-coupled cholesteryl esterase, cholesterol oxidase, and sulfated alpha-cyclodextrin. Dual-energy X-ray absorptiometry was performed using a Hologic QDR 4500A device and Apex software version 3.2 by qualified radiology technologists to assess lumbar BMD. Covariates in multivariate models may cause the correlations between HDL-C and lumbar BMD to be muddled. Age, gender, race, educational level, BMI, family income-to-poverty ratio, moderate activities, smoking at least 100 cigarettes over the life period to the point of data collection, diabetes status, hypertension status, ALT, AST, Total calcium, Cholesterol, Blood urea nitrogen, Serum uric acid, and Serum phosphorus were all covariates in this study. The NHANES website (https:// www. cdc. gov/ nchs/ nhanes/) has a thorough explanation of how these variables are calculated (Fig. 2).

### Statistical analysis

In order to account for our data set's high volatility, we used a weighted and variance estimation methodology. A weighted multivariate logistic regression model was used to investigate the connection between HDL-C and lumbar BMD. We used the weighted2 test for categorical variables and the weighted linear regression model for continuous variables to calculate the difference between each group. The subgroup analysis was carried out using stratified multivariate regression analysis. The nonlinear link between HDL-C and lumbar BMD was also addressed using smooth curve fits and generalized additive models. When nonlinearity was identified, a recursive method was used to compute the inflection point in the connection between HDL-C and BMD, followed by a two-piecewise linear regression model on both sides of the inflection point. PackageR (http://www.r-project.org) and EmpowerStats (http://www.empowerstats.com) were used for all analyses, with a P value of 0.05 deemed statistically significant. Origin (version: 2020b.https://www.originlab.com/) was used to create the HDL-C frequency distribution graph.

## Results

The individuals' weighted characteristics were divided into quartiles based on their HDL-C levels (Q1:0.16–1.03 mmol/L; Q2: 1.04–1.27 mmol/L; Q3: 1.28–1.53 mmol/L; and Q4: 1.54–4.53 mmol/L), as shown in Table 1. Our study included a total of 10,635 people between the ages of 20 and 59. Between the HDL-C quartiles, there were substantial variations in baseline characteristics. Individuals in the top HDL-C quartile were much likely to be female, whites, with lower BMI, ALT, Blood urea nitrogen, and serum uric acid and higher Income to poverty ratio, AST, Total cholesterol, Serum phosphorus, and lumbar BMD when compared to the other categories.

**Table 1:** Weighted characteristics of the study population based on high-density lipoprotein cholesterol quartiles

Direct HDL cholesterol (mmol/L)	Q1	Q2	Q3	Q4	*P* value
	(≤ 1.03)	(1.04–1.27)	(1.28–1.53)	(≥ 1.54)	
Age (years)	39.0106 ± 11.1126	38.9466 ± 11.5804	38.5032 ± 11.7201	39.9059 ± 11.9918	< 0.001
*Sex, n (%)*					< 0.001
Male	74.9792	62.7495	46.4504	30.4389	
Female	25.0208	37.2505	53.5496	69.5611	
*Race/ethnicity (%)*					< 0.001
Non-Hispanic White	59.6692	58.2902	59.6049	63.9670	
Non-Hispanic Black	8.7492	11.4206	12.3823	13.9943	
Mexican American	12.5856	12.5904	10.6419	7.0257	
Other race/ethnicity	18.9960	17.6988	17.3710	15.0130	
*Education level (%)*					< 0.001
Less than high school	18.5713	13.6541	12.9982	9.2219	
High school	24.5189	24.0084	22.4972	17.4611	
More than high school	56.9098	62.3374	64.5046	73.2764	
Body mass index (kg/m^2^)	31.8799 ± 6.7848	30.2472 ± 6.8351	28.5758 ± 6.5667	25.9654 ± 5.7135	< 0.001
Income to poverty ratio	2.6766 ± 1.6927	2.8033 ± 1.6928	2.9478 ± 1.6961	3.1525 ± 1.7327	< 0.001
*Moderate activities (%)*					< 0.001
Yes	66.1404	66.8268	71.0890	71.5667	
No	33.8596	33.1732	28.9111	28.4333	
*Smoked at least 100 cigarettes in life, n (%)*					< 0.001
Yes	47.4587	41.2512	38.1304	36.8058	
No	52.5413	58.7488	61.8696	63.1942	
*Diabetes, n (%)*					< 0.001
Yes	9.1920	6.9572	4.1151	3.0749	
No	90.8080	93.0428	95.8849	96.9251	
*Hypertension, n (%)*					< 0.001
Yes	28.8718	23.2193	20.0259	17.7677	
No	71.1282	76.7807	79.9741	82.2323	
ALT (U/L, mean ± SD)	32.4597 ± 22.4577	27.3664 ± 19.2605	23.8974 ± 17.6039	22.3850 ± 18.1097	< 0.001
AST (U/L, mean ± SD)	27.4443 ± 20.9357	24.7142 ± 16.9187	23.7748 ± 12.4046	25.1923 ± 17.0213	< 0.001
Total calcium (mg/dL, mean ± SD)	2.3392 ± 0.0849	2.3411 ± 0.0846	2.3426 ± 0.0832	2.3471 ± 0.0826	0.004
Total cholesterol (mg/dL, mean ± SD)	189.0375 ± 43.3731	189.0724 ± 40.7908	189.9795 ± 38.1432	195.3597 ± 36.1897	< 0.001
Blood urea nitrogen (mg/dL, mean ± SD)	4.6346 ± 1.5845	4.6625 ± 1.5595	4.5280 ± 1.4679	4.4968 ± 1.4788	< 0.001
Serum uric acid (umol/L, mean ± SD)	355.4584 ± 82.2140	333.1933 ± 78.9450	309.9023 ± 74.5913	285.9914 ± 72.9368	< 0.001
Serum phosphorus (mg/dL, mean ± SD)	1.1865 ± 0.1863	1.1885 ± 0.1723	1.1965 ± 0.1810	1.2208 ± 0.1765	< 0.001
Lumbar BMD (g/cm^2^, mean ± SD)	1.0320 ± 0.1466	1.0318 ± 0.1484	1.0401 ± 0.1462	1.0509 ± 0.1511	< 0.001

Mean ± SD for continuous variables: the *P* value was calculated by the weighted linear regression model

(%) for categorical variables: the *P* value was calculated by the weighted chi-square test

*BMD* bone mineral density

The findings of the multivariate regression analysis are shown in Table 2. HDL-C was positively linked with lumbar BMD in the unadjusted model (= 0.0186, 95% CI 0.0117–0.0256, P0.001). This significant correlation was remained apparent after adjusting for covariates in models 2 (= 0.0120, 95% CI 0.048–0.0193, *P* = 0.001) and 3 (= 0.0163, 95% CI 0.0084–0.0241, *P* = 0.001). Participants in the top HDL-C quartile had a 0.0127 g/cm^2^ higher BMD than those in the bottom HDL-C quartile after HDL-C had been converted from a continuous to a categorical variable (quartiles).

**Table 2:** The association between high-density lipoprotein cholesterol and lumbar bone mineral density (g/cm^2^)

	Model 1 *β* (95% CI)	Model 2 *β* (95% CI)	Model 3 *β* (95% CI)
	*P* value	*P* value	*P* value
Direct HDL cholesterol	0.0186 (0.0117, 0.0256)	0.0120 (0.0048, 0.0193)	0.0163 (0.0084, 0.0241)
	< 0.001	< 0.001	< 0.001
*Quintiles of direct HDL cholesterol*			
Q1	Reference	Reference	Reference
Q2	− 0.0002 (− 0.0083, 0.0079)	− 0.0030 (− 0.0110, 0.0050)	− 0.0036 (− 0.0115, 0.0043)
	0.962218	0.465896	0.374683
Q3	0.0081 (− 0.0002, 0.0164)	0.0024 (− 0.0059, 0.0108)	0.0020 (− 0.0065, 0.0105)
	0.057271	0.565636	0.642171
Q4	0.0190 (0.0109, 0.0270)	0.0109 (0.0026, 0.0192)	0.0127 (0.0038, 0.0216)
	< 0.001	0.010000	0.005304
*P* for trend	< 0.001	0.003	0.002
*Stratified by gender*			
Men	0.0199 (0.0083, 0.0314)	0.0089 (− 0.0024, 0.0203)	0.0160 (0.0038, 0.0282)
	< 0.001	0.123648	0.010154
Women	0.0145 (0.0052, 0.0239)	0.0150 (0.0058, 0.0242)	0.0150 (0.0048, 0.0252)
	0.002380	0.001423	0.003963
*Stratified by race*			
Non-Hispanic White	0.0113 (− 0.0000, 0.0225)	0.0070 (− 0.0050, 0.0189)	0.0119 (− 0.0013, 0.0250)
	0.050233	0.253509	0.076544
Non-Hispanic Black	0.0074 (− 0.0092, 0.0240)	0.0189 (0.0020, 0.0357)	0.0322 (0.0140, 0.0504)
	0.380599	0.028059	< 0.001
Mexican American	0.0077 (− 0.0110, 0.0263)	0.0041 (− 0.0152, 0.0233)	− 0.0040 (− 0.0250, 0.0170)
	0.419084	0.679443	0.708012
Other race	0.0212 (0.0078, 0.0345)	0.0263 (0.0120, 0.0406)	0.0286 (0.0132, 0.0440)
	0.001878	< 0.001	< 0.001

Model 1: no covariates were adjusted. Model 2: age, gender, and race were adjusted. Model 3: age, gender, race, educational level, BMI, family income-to-poverty ratio, moderate activities, smoking status, diabetes status, hypertension status, ALT, AST, total calcium, cholesterol, blood urea nitrogen, serum uric acid, and serum phosphorus were adjusted

In the subgroup analysis stratified by gender and race, the model is not adjusted for sex and race, respectively

**Fig. 3:**
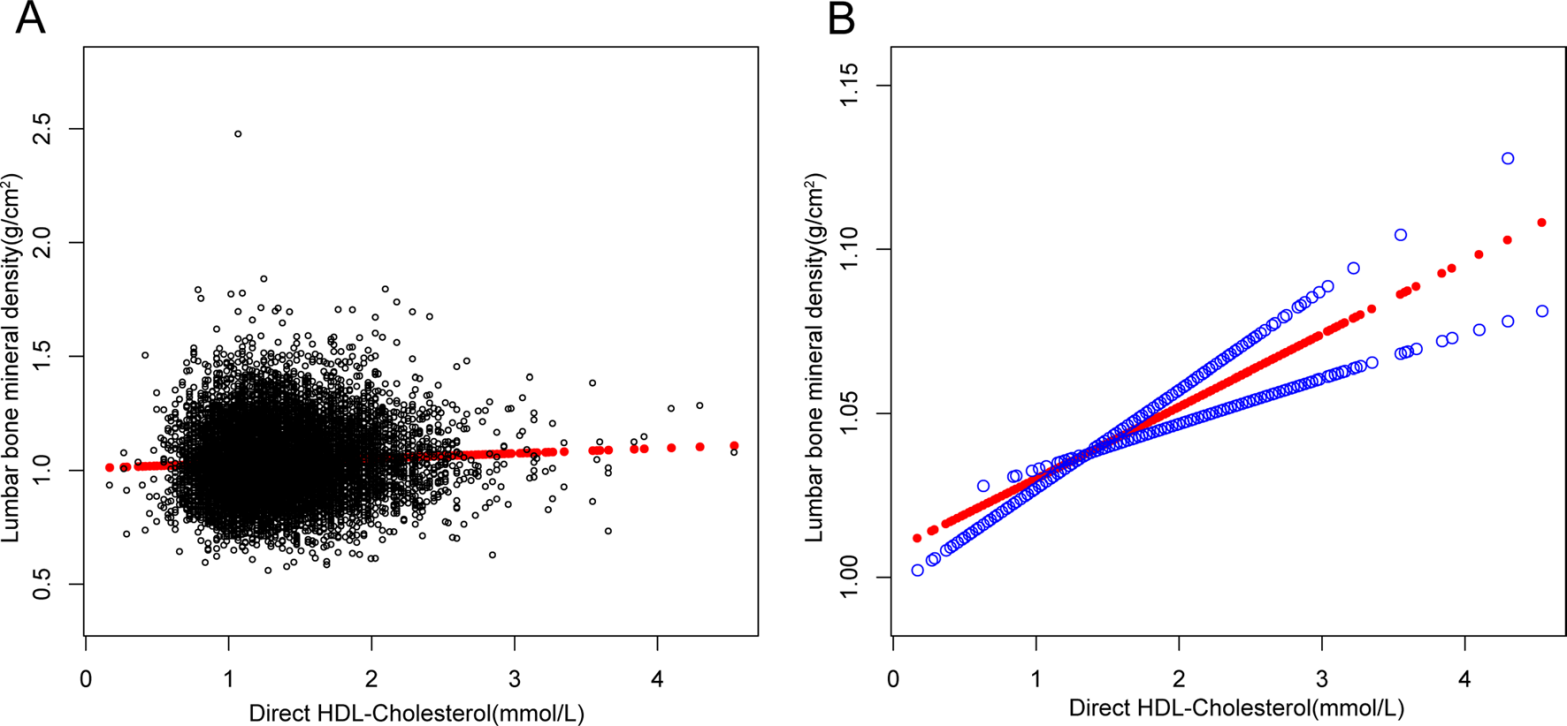
The association between high-density lipoprotein cholesterol and lumbar bone mineral density. **a** Each black point represents a sample. **b** Solid redline represents the smooth curve fit between variables. Blue bands represent the 95% of confidence interval from the fit. Age, gender, race, educational level, *BMI* family income-to-poverty ratio, moderate activities, smoking at least 100 cigarettes over the life period to the point of data collection, diabetes status, hypertension status, *ALT AST* total calcium, Cholesterol, Blood urea nitrogen, Serum uric acid, and Serum phosphorus were adjusted

**Fig. 4:**
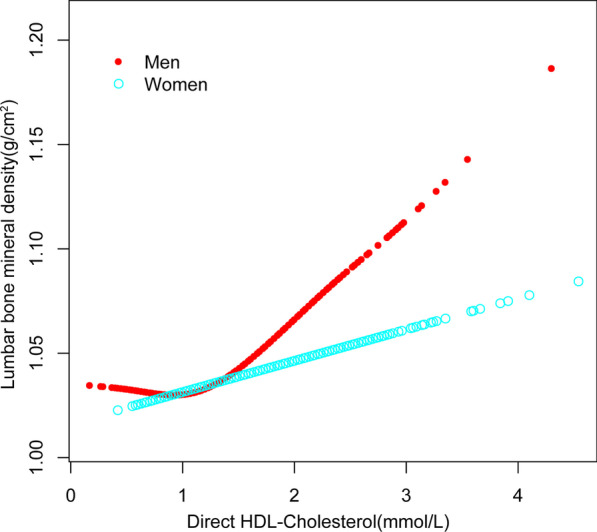
The association between high-density lipoprotein cholesterol and lumbar bone mineral density stratified by sex. Age, race, educational level, *BMI* family income-to-poverty ratio, moderate activities, smoking at least 100 cigarettes over the life period to the point of data collection, diabetes status, hypertension status, *ALT* AST, Total calcium, Cholesterol, Blood urea nitrogen, Serum uric acid, and Serum phosphorus were adjusted

**Fig. 5:**
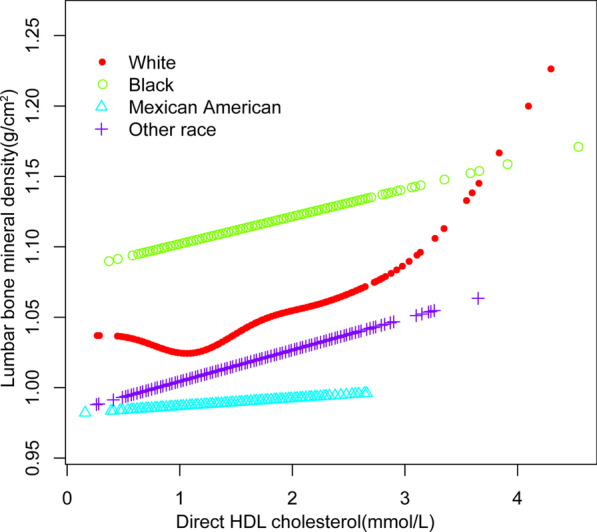
The association between high-density lipoprotein cholesterol and lumbar bone mineral density stratified by race. Age, gender, educational level, BMI, family income-to-poverty ratio, moderate activities, smoking at least 100 cigarettes over the life period to the point of data collection, diabetes status, hypertension status, ALT, AST, Total calcium, Cholesterol, Blood urea nitrogen, Serum uric acid, and Serum phosphorus were adjusted

The beneficial correlation of HDL-C with lumbar BMD maintained in women (= 0.0150, 95% CI 0.0048–0.0252, *P* = 0.004), but not in men (= 0.0160, 95% CI 0.0038–0.0282, *P* = 0.010), as well as in blacks (= 0.0322, 95% CI 0.0140–0.0504, P < 0.001), but not in whites (= 0.0322, 95% CI 0.0140–0.0504, *P* = 0.077). Figures 3, 4 and 5 depict smooth curve fits and generalized additive models that were utilized to define the nonlinear connection between HDL-C and lumbar BMD. The relationship between HDL-C and lumbar BMD in males was a U-shaped curve at 0.98 mmol/L for HDL-C (Table 3). The relationship between HDL-C and lumbar BMD in whites was likewise a U-shaped curve, with a level of 0.98 mmol/L for HDL-C (Table 4).

**Table 3:** Threshold effect analysis of high-density lipoprotein cholesterol on lumbar bone mineral density in men using the two-piecewise linear regression model

Lumbar bone mineral density	Adjusted β **(**95% CI) *P* value
Men	
Fitting by the standard linear model	0.0160 (0.0038, 0.0282)
Fitting by the two-piecewise linear model	0.0102
Inflection point	0.98
Direct HDL cholesterol < 0.98(mmol/L)	− 0.1302 (− 0.1875, − 0.0729)
	< 0.0001
Direct HDL cholesterol > 0.98(mmol/L)	0.0341 (0.0201, 0.0481)
	< 0.0001
Log likelihood ratio	< 0.001

Age, gender, race, educational level, BMI, family income-to-poverty ratio, moderate activities, smoking status, diabetes status, hypertension status, ALT, AST, total calcium, cholesterol, blood urea nitrogen, serum uric acid, and serum phosphorus were adjusted

**Table 4:** Threshold effect analysis of high-density lipoprotein cholesterol on lumbar bone mineral density in whites using the two-piecewise linear regression model

Lumbar bone mineral density	Adjusted *β* **(**95% CI) *P* value
Whites	
Fitting by the standard linear model	0.0095 (− 0.0036, 0.0225)
Fitting by the two-piecewise linear model	0.1560
Inflection point	0.98
Direct HDL cholesterol < 0.98(mmol/L)	− 0.1812 (− 0.2666, − 0.0959)
	< 0.0001
Direct HDL cholesterol > 0.98(mmol/L)	0.0220 (0.0079, 0.0362)
	0.0023
Log likelihood ratio	< 0.001

Age, gender, race, educational level, BMI, family income-to-poverty ratio, moderate activities, smoking status, diabetes status, hypertension status, ALT, AST, total calcium, cholesterol, blood urea nitrogen, serum uric acid, and serum phosphorus were adjusted

## Discussion

Our multivariate logistic regression analysis revealed that a higher HDL-C level was associated with a higher lumbar BMD in the current research. On subgroup analysis, however, we discovered a U-shaped relationship between HDL-C and lumbar BMD in males and whites. It's worth noting that males and whites have the same inflection point at 0.98(mmol/L).

HDL-C is well known for its ability to promote reverse cholesterol transport and thus reduce the chance of developing cardiovascular disease, in addition to being associated with a variety of chronic diseases [24, 25], such as chronic kidney disease [26], obesity [27], diabetes [28], endocrine disorders, and metabolic syndrome [29–32]. On the other hand, HDL-C may have a role in osteoporosis, since a higher HDL-C was linked to a higher lumbar BMD in most people in our representative US sample. Given this link, HDL-C might serve as a possible osteoporosis biomarker. As a result, measuring HDL-C levels might be used as a screening tool for osteoporosis, guiding treatment measures and avoiding HDL-C overcorrection in osteoporosis patients.

Clinical investigations on the connection between HDL-C and BMD in adults are still few and disputed. In three cross-sectional studies conducted in China, researchers discovered a negative relationship between higher HDL-C and higher BMD in people aged 20–59 [18, 19, 33]. Other research from Korea and Iran [20, 21], as well as from the United States [34], backed up this result. However, other studies contradicted this finding. A cross-sectional research conducted in Spain found a favorable connection between HDL-C and BMD in postmenopausal women (*n* = 667) [22]. A cross-sectional and longitudinal research from Korea found a small but significant positive connection between femur neck BMD and HDL-C in postmenopausal women (*n* = 958) (correlation coefficient, 0.077; 95% confidence interval, 0.005–0.149) [35]. Furthermore, a cross-sectional research with 481 Chinese seniors found no evidence of a causal relationship between HDL-C and BMD at various locations [23].

The current study not only shows a link between HDL-C and BMD, but it also has therapeutic implications that can help doctors. The positive correlation shows that people with a higher HDL-C level may also have a higher BMD. We also ran subgroup analysis in our research for a more accurate depiction of the data set. Our findings suggest that in both men and women, and either race, higher HDL-C is associated with higher lumbar BMD when HDL-C is greater than 0.98 mmol/L, and this association is more pronounced in men, whereas in men as well as whites when HDL-C is less than 0.98 mmol/L, the relationship among HDL-C levels and lumbar BMD is negatively correlated. Furthermore, stratified by sex and ethnicity, our subgroup analyses revealed, for the first time, a U-shaped relationship between HDL-C and lumbar BMD among men of the same race as whites. Gender and race inequalities might be explained by variations in estrogen levels, genetic risk factors, environmental risk factors, obese status, and other variables. To further understand the link between HDL-C and BMD by gender and race, more prospective studies with large study populations are needed.

As a chronic illness, osteoporosis needs long-term management and therapy. This is reflected in both nutritional intake and pharmacological treatment [3]. A recent study suggests that higher dietary protein intake may reduce fracture risk when calcium is in adequate supply, and dairy products are an important source of both nutrients. In addition, a balanced diet, which includes minerals, protein, fruits and vegetables, is important for bone health and the prevention of fragility fractures [36]. Also, our study provides some references for the amount of daily dietary cholesterol intake in the population. The general objective of osteoporosis treatment is to lower the risk of fracture, and the absolute reduction in fracture risk is dependent on the individual's risk as well as the medicine used [37]. Bisphosphonates are the most commonly used drugs in the treatment and prevention of osteoporosis, alendronate, risedronate, and zoledronic acid are all beneficial at lowering the risk of vertebral, nonvertebral, and hip fractures [38]. Treatment of osteoporosis does not include the administration of non-steroidal anti-inflammatory drugs (NSAIDs), but the pain associated with osteoporotic fractures is often treated with the administration of NSAIDs [39]. But when treating osteoporosis and managing the associated pain, physicians also need to pay enough attention to avoid drug-drug interactions in adverse drug reactions. A recent study mined several known interactions from an electronic health records (EHRs) dataset of approximately 400,000 inpatients through a machine learning algorithm of logistic regression and pointed to a new, potentially hepatotoxic interaction that may occur with the concomitant use of meloxicam and esomeprazole. This will undoubtedly deepen the understanding of physicians in drug-drug interactions [40].

The mechanisms that explain the link among HDL-C and BMD remain unknown. There is no strong evidence to support this detrimental relationship, especially in fundamental studies. According to related study, there are several possible explanations for this phenomenon. First, HDL-C affects BMD through estrogen. It is generally known that after menopause, the risk of cardiovascular disease rises considerably in females [41]. In addition, during the peri-menopausal phase, BMD begins to diminish and bone resorption rates rise in females [42]. Menopause is thought to be the cause of an increase in cardiovascular disease and a reduction in BMD, at least in part, by the lack of estrogen. Estrogen has a significant biological effect on lipid metabolism in the blood and bone resorption regulation [41, 43]. In postmenopausal women, the use of hormone replacement therapy (HRT) has been connected to an increase in HDL and BMD levels [44, 45]. As a result, estrogen level may have an impact on the link among HDL and BMD in women. Second, HDL has the power to influence the bone's osteoblasts and osteoclasts directly. In investigations using these cells, the capacity of osteoblast-like cell lines to internalize and destroy particular subclasses of HDL particles was established. Although osteoblasts have been shown to selectively absorb cholesterol esters from HDL, it is unknown whether this absorption is mediated by the Scavenger receptor class B type I, scavenger receptor class B type II, and CD36 cell surface receptors [46]. Third, Heritable variables are thought to account for up to 85% of the variance in BMD, whereas heritability predictions for blood HDL levels vary from 40 to 60% [47, 48]. The lack of agreement among research looking at the link between HDL and BMD is most likely due to genetic variations in the study groups [49]. Furthermore, studies have shown that BMD and HDL levels are affected by gene-environment interactions [50–55].

Postmenopausal women have been the focus of the majority of cohort and cross-sectional research so far. The link between HDL and BMD in young healthy people is poorly known. The findings of our study are extremely applicable to the entire population since we selected a nationally comprehensive sample. Furthermore, because of our large sample size, we were able to conduct subgroup analyses of HDL-C and lumbar BMD in people of different genders and races. However, it is critical to recognize the study's limitations. The cross-sectional methodology of our investigation, first and foremost, restricts the inference of a causal relationship between HDL-C and lumbar BMD in adults. More fundamental mechanistic research and large sample prospective studies are needed to understand the particular mechanism of the link among HDL-C and BMD. Second, malignancy patients were excluded from the research because cancer might have a big impact on lumbar BMD. Third, because information on sex hormone levels was not accessible or absent from the NHANES database 2011–2018, our study was unable to explain these situations in the present patients.

## Conclusion

In people aged 20 to 59, our research discovered a positive association among HDL-C and lumbar BMD. Among males and whites, this relationship followed a U-shaped curve (inflection point: 0.98 mmol/L). HDL-C measurement might be used as a responsive biomarker for detecting osteoporosis early and guiding therapy.

## Data Availability

The survey data are publicly available on the Internet for data users and researchers throughout the world ( www.cdc.gov/nchs/nhanes/).

## Abbreviations

HDL: High-density lipoproteins
HDL-C: High-density lipoprotein cholesterol
CVDs: Cardiovascular diseases
BMD: Bone mineral density
NHANES: National Health and Nutrition Examination Survey
apoB: Apoprotein-B
EHRs: Electronic health records
NSAIDs: Non-steroidal anti-inflammatory drugs
